# Clinical relevance of critical plasma homocysteine levels in predicting rupture risk for small and medium-sized intracranial aneurysms

**DOI:** 10.1038/s41598-024-69219-4

**Published:** 2024-08-06

**Authors:** Wang Lu, Yan Shiwei, Li Aimin, Xie Kang

**Affiliations:** 1 Department of Neurosurgery, Affiliated Lianyungang Hospital of Xuzhou Medical University, Lianyungang, 222002 Jiangsu China; 2 Jinzhou Medical University, Jinzhou, China

**Keywords:** Intracranial aneurysms, Plasma homocysteine, Rupture risk, Critical concentrations, Vascular wall damage, Independent risk factor, Prognosis, Neurological disorders, Neurological disorders

## Abstract

Plasma homocysteine (Hcy) has been globally recognized as an independent risk factor for various neurovascular diseases. In this study, the authors investigated the relationship between critical Hcy concentration and the risk of rupture in intracranial aneurysms (IAs). This study collected data from 423 patients with both ruptured and unruptured IAs. We compared demographic data, vascular rupture risk factors, and laboratory test results between the two groups. Multivariable logistic regression analysis was employed to determine the correlation between critical plasma Hcy levels and the risk of rupture in small to medium-sized IAs. A total of 330 cases of ruptured intracranial aneurysms (RIA) and 93 cases of unruptured intracranial aneurysms (UIA) were included. Univariate analysis revealed statistically significant differences between the ruptured and unruptured groups in terms of hypertension, hyperlipidemia, plasma Hcy levels, and IA morphology (all *P* < 0.05). Multivariable logistic regression analysis indicated that hypertension (odds ratio [*OR*] 0.504; 95% confidence interval [*CI*] 0.279–0.911;* P* = 0.023), hyperlipidemia (*OR* 1.924; 95% *CI* 1.079–3.429;* P* = 0.027), and plasma Hcy levels (*OR* 1.420; 95% *CI* 1.277–1.578; *P* < 0.001) were independently associated with the rupture of small to medium-sized IAs, all with statistical significance (*P* < 0.05). Our study suggests that critical plasma Hcy levels are an independent risk factor for increased rupture risk in small to medium-sized intracranial aneurysms. Therefore, reducing plasma Hcy levels may be considered a valuable strategy to mitigate the risk of intracranial vascular abnormalities rupture and improve patient prognosis.

## Introduction

Intracranial aneurysms (IAs) refer to the localized bulging of arterial walls resulting from structural disruption of the internal elastic lamina and media, accompanied by hemodynamic abnormalities. The overall prevalence of IAs in the population is approximately 5%, with unruptured intracranial aneurysms (UIA) accounting for around 1%^1^. Epidemiological data suggest an annual rupture rate of IAs at about 1%^2^, indicating that the majority of IAs remain asymptomatic or go undetected throughout an individual’s life. However, studies indicate that the acute rupture fatality rate of UIA can reach 30%^3^, with a 40% rate of re-bleeding within one month post-rupture and mortality exceeding 40%^4^. Consequently, IAs rupture is a primary cause of heightened mortality risk, increased life burden, and compromised patient prognosis. Consequently, it is imperative to explore and intervene in relevant risk factors to prevent the formation and rupture tendency of IAs.

While the impact of conventional risk factors has been comprehensively studied^5,6^, the global IAs rupture rate continues to increase annually even with these factors effectively managed and controlled in clinical settings. Thus, these factors can only partially explain the reasons behind the heightened risk of IAs rupture. This has propelled the study of biomarkers’ modifiability into a key research domain. Recent findings have increasingly reported a novel vascular-inflammatory risk factor—homocysteine (Hcy)—which has been associated with atherosclerosis, stroke, dementia, and the occurrence of various neurological vascular diseases^7,8^. Numerous studies have affirmed the connection between Hcy and the development of IAs^9,10^. According to previous standards, high homocysteine levels (HHcy) are characterized by elevated blood Hcy levels and classified as mild (15–30 μmol/L), intermediate (31–100 μmol/L), and severe (> 100 μmol/L). Prolonged HHcy can lead to multi-system damage in the nervous, cardiovascular, renal, ocular, and skeletal domains^11^. Given the lack of specific clinical manifestations, low survival rates, and high re-bleeding rates, both domestic and international experts emphasize its pathological role. Various guidelines have previously noted that plasma Hcy levels ≥ 15 μmol/L are an independent risk factor for the occurrence of cardiovascular and cerebrovascular diseases. However, related international consensus suggests that plasma Hcy levels need not rise to exceedingly high levels to exert detrimental effects on blood vessels. Recent discoveries indicate that even when plasma Hcy levels reach 6.3 μmol/L, individuals enter a high-risk zone for cardiovascular events, and at a level of 10 μmol/L, the rate of cardiovascular events doubles^12^.

Although small aneurysms are considered less likely to rupture^13^, they are still prone to rupture. The size of intracranial aneurysms (IAs) has been identified as a predictor of aneurysm instability and rupture risk, with a commonly accepted rupture risk threshold of 5–10 mm^14,15^. Generally, aneurysms smaller than 7 mm are thought to have a low likelihood of rupture^16,17^. However, recent retrospective studies have observed a higher-than-expected incidence of ruptures in small aneurysms. In response, our study adopts a novel approach by examining the influence of two critical factors: plasma homocysteine concentration and IA diameter < 15 mm. By integrating these factors, we aim to enhance our understanding of how critical plasma homocysteine levels can predict the rupture risk of small to medium-sized IAs.

## Methods

### Participant selection criteria

In this case–control study, we included a total of 330 patients with RIAs and 93 patients with UIAs, who were admitted to the hospital between January 2018 and July 2022. All patients were diagnosed using computed tomography angiography (CTA) or digital subtraction angiography (DSA).

### Inclusion criteria

(1) Age ≥ 18 years; (2) Patients diagnosed with IAs based on CTA or DSA imaging, as per World Health Organization criteria, with a maximum confirmed IAs diameter ≤ 15 mm^18^; (3) Patients with plasma homocysteine (Hcy) levels ≤ 15 μmol/L upon admission.

### Exclusion criteria

(1) Patients who received medications such as folic acid or vitamin B_12_ within the last 3 months that could dynamically affect plasma Hcy levels; (2) Patients with IAs rupture due to rare causes like cerebral vascular malformations or trauma; (3) Patients who did not undergo blood index measurements upon admission, including lipids and glucose; (4) Cases of severe systemic diseases leading to patient death.

### Data collection and laboratory measurements

Two neurosurgery specialists retrospectively collected baseline data including patient age, gender, hypertension, hyperlipidemia, diabetes, plasma Hcy levels, smoking status, alcohol consumption, and history of stroke. All patients underwent cranial imaging (CTA, DSA) to identify the presence of IAs and assess morphological risk factors related to aneurysm rupture (including location, maximum size, aneurysm neck size, and shape). The size of IAs was recorded based on the maximum diameter. Patients were included in the study if any of these imaging examinations confirmed the presence of IAs and their size met the study requirements. The IAs patients were categorized into the UIA group (n = 93) if diagnosed with unruptured IAs and the RIA group (n = 330) if diagnosed with ruptured IAs via imaging without recent use of relevant medications, vitamins, folic acid, antiepileptics, or other substances that might affect plasma Hcy levels. The critical plasma Hcy concentration range was defined as 8.9 μmol/L ≤ Hcy ≤ 15 μmol/L. Definitions for hypertension, hyperlipidemia, diabetes, stroke history, alcohol consumption, and smoking were standardized based on established clinical criteria.

### Statistical analysis

Data processing was performed using SPSS 24.0 statistical software and graphical representations were created using *R* programming language. All analyses were conducted using a two-tailed probability test (*P* < 0.05) to assess statistical significance.

(1) The Shapiro–Wilk test was utilized to assess the normal distribution of data in the two groups. (2) Statistical differences between the two groups were assessed. Normally distributed continuous variables were expressed as mean ± standard deviation (mean ± SD), and independent samples Student’s t-test was employed for between-group comparisons. (3) In cases where normality assumptions were violated, the Mann–Whitney U test was used for continuous variables, presenting results as median and interquartile range [M (P25, P75)]. (4) Categorical variables were presented as frequencies or percentages (%) and analyzed using chi-square test or Fisher’s exact test. (5) Univariate analysis identified risk factors (*P* < 0.05) related to IAs rupture, which were then included as candidate variables in a multivariable logistic regression model to determine independent risk factors affecting IAs rupture. Adjusted and unadjusted OR with 95% CI was reported to quantify the association strength between critical plasma Hcy concentration and small-to-medium-sized IAs rupture. (6) Stratification based on plasma Hcy concentration levels was performed, and Spearman correlation analysis was employed to investigate the relationship between critical plasma Hcy levels and small-to-medium-sized IAs rupture. (7) Interaction and stratification analyses were conducted, primarily considering variables such as age, gender, hypertension, diabetes, hyperlipidemia, ischemic stroke, hemorrhagic stroke, smoking, and alcohol consumption status.

### Ethical approval

The research conducted for this study received ethical approval from the Affiliated Lianyungang Hospital’s Ethics Committee. The committee is responsible for overseeing the ethical aspects of research involving human participants, ensuring that the study adheres to established guidelines and regulations. The research followed the ethical guidelines outlined by the Declaration of Helsinki for research involving human participants. For the publication of study results, additional informed consent was obtained from participants. They were informed about the nature of the publication and any identifiable information that might be included. Confidentiality was maintained, and any personal identifiers were removed to ensure privacy.

## Results

**Figure 1:**
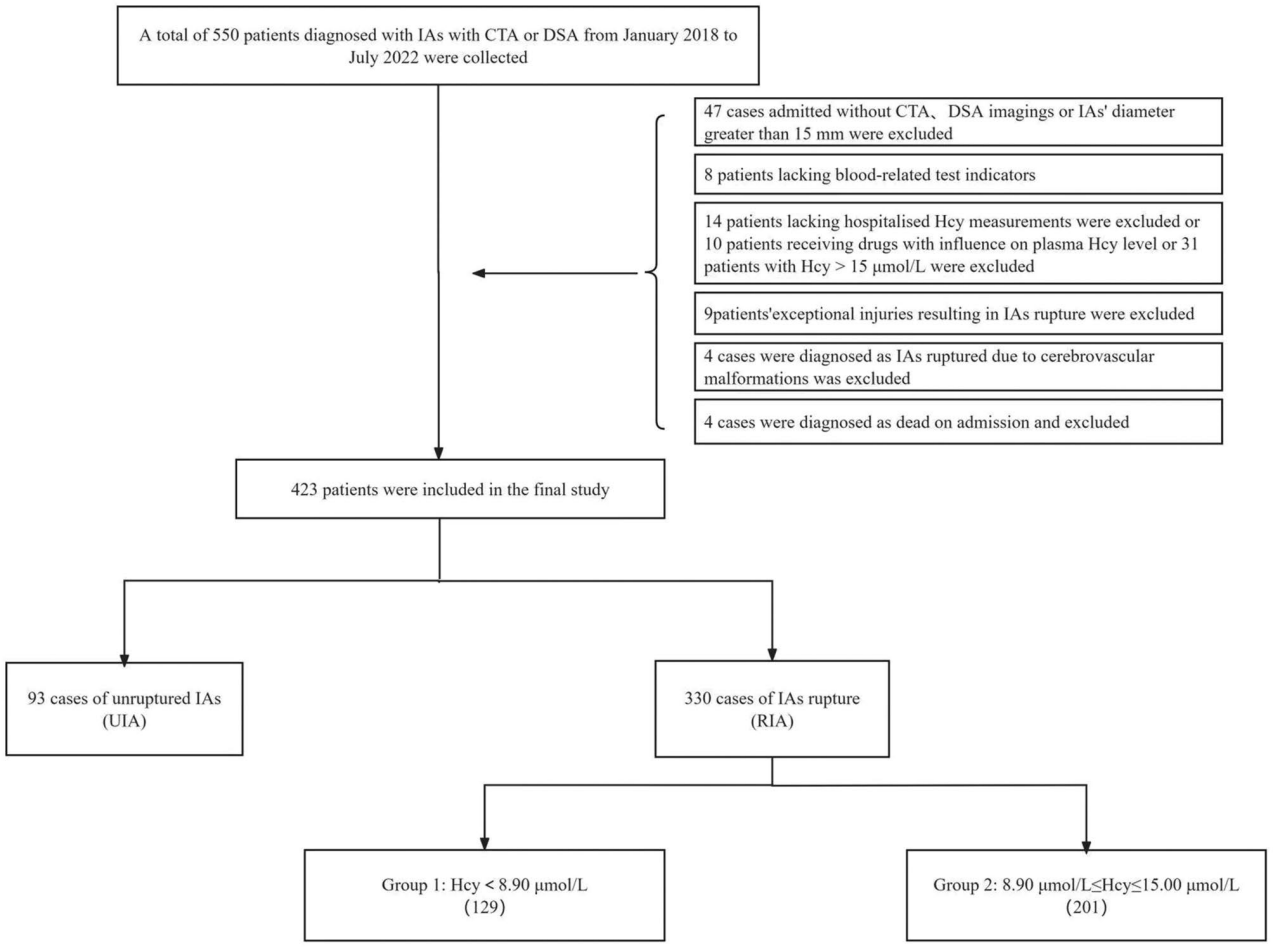
Data collection flow chart.

A total of 550 patients diagnosed with IAs via DSA or CTA from January 2018 to July 2022 were collected. Among them, 127 cases were excluded: 100 lacked relevant imaging or admission blood index results, 10 were on medications affecting plasma Hcy levels, 13 experienced IAs rupture due to other reasons, and 4 exhibited clinical outcomes of death. Eventually, 423 cases were included, with 93 classified as UIAs and 330 as RIAs. Within the RIA group, patients were further stratified based on plasma Hcy levels into Group 1 (Hcy < 8.9 μmol/L) and Group 2 (8.9 μmol/L ≤ Hcy ≤ 15 μmol/L) (Fig. 1).

Population demographics and conventional vascular risk factors of the participants are presented in Table 1. Among the 423 included patients, the average age was 58.56 ± 11.62 years, with 33.1% being male. UIA and RIA exhibited minor differences in age, with a higher prevalence among females (66.7% *vs* 33.3%). RIA patients had higher rates of hypertension (82.7% *vs* 17.3%, *P* = 0.022) and hyperlipidemia (51.2% *vs* 48.8%, *P* = 0.047). The RIA group had significantly higher Hcy levels (median: 9.50 μmol/L, interquartile range: 7.40–11.72 μmol/L) compared to the UIA group (median: 7.09 μmol/L, interquartile range: 5.40–8.50 μmol/L, *P* < 0.001), along with a distinct association with IAs morphology (80.9% *vs* 19.1%, *P* = 0.046) (Table 1).

**Table 1:** Demographic and clinical characteristics.

Baseline feature	Total (n = 423)	RIA group	UIA group	*P-*value
Age (years)	58.56 ± 11.62	58.57 ± 11.78	58.53 ± 11.09	0.442
Sex (Male)	140 (33.1%)	110 (33.3%)	30 (32.3%)	0.846
Hypertension, yes	340 (80.4%)	273 (82.7%)	67 (72.0%)	0.022*
Diabetes, yes	48 (11.3%)	41 (12.4%)	7 (7.5%)	0.188
hyperlipidemia, yes	212 (50.1%)	169 (51.2%)	43 (46.2%)	0.047*
Ischemic stroke, yes	23 (5.4%)	15 (4.5%)	8 (8.6%)	0.128
Hemorrhagic stroke, yes	15 (3.5%)	9 (2.7%)	6 (6.5%)	0.086
Smoking, yes	64 (15.1%)	53 (16.1%)	11 (11.8%)	0.314
Drinking, yes	52 (12.3%)	45 (13.6%)	7 (7.5%)	0.113
Homocysteine (μmol/L)	8.90 (6.50–10.90)	9.50 (7.40–11.72)	7.09 (5.40–8.50)	< 0.001*
Aneurysmal features				
Aneurysm location				0.437
Anterior communicating aneurysm	153 (36.2%)	113 (34.2%)	40 (43.0%)	
Middle cerebral aneurysm	122 (28.8%)	91 (27.6%)	31 (33.3%)	
Posterior communicating aneurysm	148 (35.0%)	126 (38.2%)	22 (23.7%)	
Aneurysm size (mm)	4.81 (3.20–5.70)	4.84 (3.20–5.70)	4.69 (3.30–5.60)	0.849
Aneurysmal neck size (mm)	2.59 (1.70–3.20)	2.67 (1.80–3.20)	2.31 (1.20–3.05)	0.087
Number of aneurysms				0.433
1	383 (90.5%)	302 (91.5%)	81 (87.1%)	
2	37 (8.7%)	26 (7.9%)	11 (11.8%)	
3	3 (0.8%)	2 (0.6%)	1 (1.1%)	
Intracranial aneurysm shape				0.046*
Saccular aneurysm	351 (83.0%)	267 (80.9%)	84 (90.3%)	
Fusiform aneurysm	68 (16.1%)	59 (17.9%)	9 (9.7%)	
Irregular aneurysm	4 (0.9%)	4 (1.2%)	0 (0%)	

*Indicates statistically significant difference compared to the reference group or control group (*P* < 0.05).

**Figure 2:**
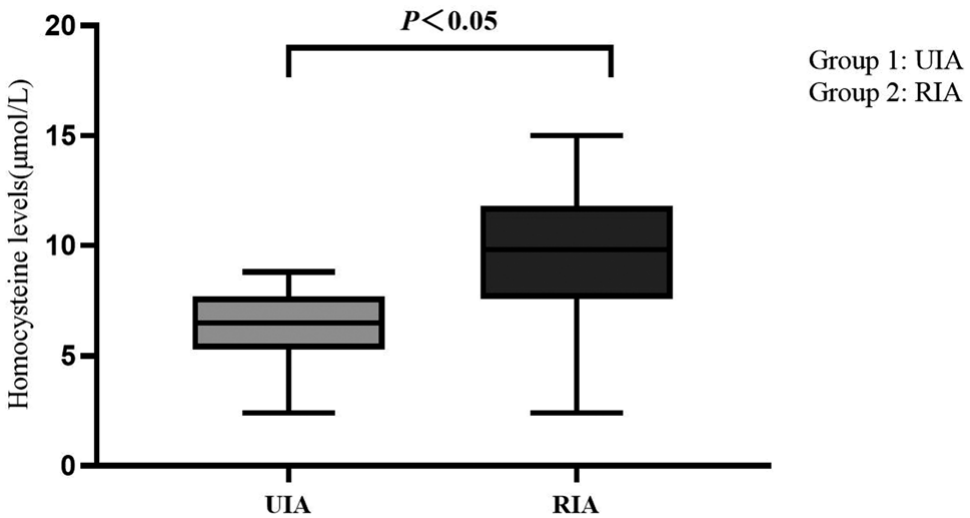
Distribution of homocysteine levels in two groups.

No significant differences were observed between RIA and UIA groups regarding age, gender, diabetes, history of stroke (including ischemic and hemorrhagic stroke), smoking, alcohol consumption status, as well as aneurysm location, maximum diameter, and quantity (*P* > 0.05). Plasma Hcy levels for UIA and RIA patients are presented in Table 2, with RIA patients exhibiting significantly higher serum Hcy levels of 9.50 μmol/L (7.40–11.72 μmol/L) compared to UIA patients with 7.09 μmol/L (5.40–8.50 μmol/L), *P* < 0.05 (Fig. 2).

**Table 2:** Univariate logistic regression model analysis to assess risk factors associated with IAs rupture.

Variable	B	OR	95% CI	*P-*value
Hypertension	0.600	0.504	0.279–0.911	0.023
Hyperlipidemia	0.654	1.924	1.079–3.429	0.027
Homocysteine (μmol/L)	0.350	1.420	1.277–1.578	0.000
Intracranial aneurysm shape	− 19.553	0.000	–	> 0.05

Risk factors meeting the criterion of *P* < 0.05 among conventional rupture risk factors were included as independent variables for multivariable logistic regression analysis. This analysis revealed that hypertension (odds ratio [*OR*] 0.504; 95% confidence interval [*CI*] 0.279–0.911;* P* = 0.023), hyperlipidemia (*OR* 1.924; 95% *CI* 1.079–3.429;* P* = 0.027), and plasma Hcy levels (*OR*: 1.420; 95% *CI* 1.277–1.578; *P* < 0.001) were significantly independently associated with IAs rupture (Table 2). However, the morphology of intracranial aneurysms did not show significant independent correlation with aneurysm rupture (*P* > 0.05).

To account for confounding effects, a multivariable logistic regression model with IAs rupture as the outcome variable was designed. The strength of the association between Hcy and IAs was quantified, and unadjusted and adjusted odds ratios (ORs) with 95% CI were reported. The results indicated that the critical plasma Hcy concentration (8.9 μmol/L ≤ Hcy ≤ 15 μmol/L) was significantly associated with small and middle-sized IAs rupture (OR 2.314, 95% CI 1.390–3.852, *P* < 0.001) (Table 3). Further stratified analysis of RIA patients based on the average plasma Hcy concentration of the overall population and Spearman correlation analysis revealed that the critical level of plasma Hcy was an independent risk factor for small and middle-sized IAs rupture (Table 4).

**Table 3:** Association between homocysteine and intracranial aneurysm evaluated by multivariable logistic regression analysis.

Variable	No. (%) of study participants		95% CI	
	RIA group	UIA group	Crude	Adjust^a^
Homocysteine (μmol/L)	93	330	1.405 (1.271–1.554)	1.467 (1.303–1.653)
Hcy < 8.9 μmol/L	73 (78.5%)	129 (45.2%)	*P* > 0.05	*P* > 0.05
8.9 μmol/L ≤ Hcy ≤ 15 μmol/L	20 (21.5%)	201 (54.8%)	1.878 (1.238–2.849)	2.314 (1.390–3.852)

^a^Adjusted for Hypertension, Hyperlipidemia, and IAs shape.

**Table 4:** Spearman correlation analysis of the relationship between homocysteine and intracranial aneurysm.

	Hcy	Broken outcome
Spearman’s rho		
Hcy		
Correlation coefficient	1.000	0.327**
Sig(2-tailed)		0.000
N	423	423
Broken outcome		
Correlation coefficient	0.327**	1.000
Sig(2-tailed)	0.000	
N	423	423

**Indicates statistically significant difference compared to the reference group or control group (*P* < 0.05).

Table 5, utilizing stratification and interaction analysis, confirmed the association between critical Hcy concentration and IAs rupture risk. Apart from the stratified factors themselves, adjustments were made for all factors including age, gender, hypertension, hyperlipidemia, diabetes, hemorrhagic events, ischemic events, smoking, and alcohol status in each stratum. The consistency of the association between critical Hcy concentration and IAs rupture was observed in both stratified and multivariable logistic regression analyses. Stratified analysis showed that patients aged 58 years or older (adjusted OR 2.794, 95% CI 1.183–6.601), males (adjusted OR 1.703, 95% CI 1.339–2.166), those with hypertension (adjusted OR 1.464, 95% CI 1.147–1.869), hyperlipidemia (adjusted OR 2.405, 95% CI 0.094–6.395), or diabetes (adjusted OR 1.457, 95% CI 0.934–2.271), smokers (adjusted OR 1.781, 95% CI 1.131–2.805), and long-term alcohol consumers (adjusted OR 2.737, 95% CI 1.081–6.929) exhibited statistically significant associations with IAs rupture. The results of interaction analysis indicated that no apparent interacting factors were influencing the association between critical Hcy concentration and IAs rupture (Table 5).

**Table 5:** Association between critical plasma concentrations of Hcy and IAs rupture based on baseline characteristics.

Subgroup	RIA group		UIA group		OR (95% CI)		*P*-value for interaction
	Safe concentration	Critical concentration	Safe concentration	Critical concentration	Crude	Adjust^a^	
Age							0.334
< 58	77	73	10	34	1.275 (1.115–1.457)	1.075 (0.484–2.386)	
≥ 58	114	66	10	39	1.547 (1.329–1.800)	2.794 (1.183–6.601)	
Sex							0.911
Male	76	34	9	21	1.552 (1.281–1.881)	1.703 (1.339–2.166)	
Female	125	95	11	52	1.361 (1.207–1.534)	1.405 (1.227–1.609)	
Hypertension							0.954
Yes	165	108	13	54	1.433 (1.271–1.615)	1.464 (1.147–1.869)	
No	36	21	7	19	1.362 (1.121–1.655)	1.446 (1.284–1.674)	
Diabetes							0.772
Yes	31	10	1	6	1.402 (1.036–1.898)	1.457 (0.934–2.271)	
No	170	119	19	67	1.402 (1.260–1.559)	1.448 (1.291–1.624)	
Hyperlipidemia							0.600
Yes	103	66	7	36	1.194 (0.921–1.548)	2.405 (0.094–6.395)	
No	94	67	12	38	1.338 (1.173–1.527)	0.884 (0.315–2.467)	
Ischemic stroke							0.737
Yes	103	66	2	6	1.416 (0.903–2.220)	Ref	
No	86	75	18	67	1.409 (1.207–1.563)	1.448 (1.291–1.624)	
Hemorrhagic stroke							0.209
Yes	6	3	0	6	3.182 (0.883–11.463)	Ref	
No	195	126	20	67	1.383 (1.250–1.531)	1.426 (1.274–1.597)	
Smoking							0.335
Yes	19	34	3	8	1.452 (1.094–1.927)	1.781 (1.131–2.805)	
No	167	110	17	65	1.397 (1.254–1.555)	1.448 (1.283–1.634)	
Drinking							0.996
Yes	32	13	1	6	1.398 (1.018–1.919)	2.737 (1.081–6.929)	
No	169	116	19	67	1.402 (1.261–1.559)	1.442 (1.282–1.621)	

*Each stratification adjusted for all factors (age, sex, hypertension, diabetes, hyperlipidemia, bleeding events, ischemic events, smoking status and drinking status) except the stratification factor itself.

## Discussion

Arterial aneurysmal subarachnoid hemorrhage (aSAH) is the most destructive subtype of stroke, with a mortality rate of approximately 50% among survivors^13^. The majority of cases are attributed to the rupture of intracranial aneurysms (IAs), occurring at a relatively young age, with an average around 50 years old. About one-third of patients die shortly after the rupture, and survivors often experience varying degrees of lifelong neurological dysfunction or even disability. Only 5% of patients can fully recover to normal work and life^14^. Considering the catastrophic consequences of IAs rupture and the lack of effective pharmacological interventions, despite rapid advancements in surgical techniques and auxiliary devices, the treatment outcomes remain suboptimal. Many neurosurgeons believe that, rather than attempting to improve clinical outcomes after the damage has occurred, effectively preventing the formation and rupture of IAs seems to be a more ideal treatment direction. Therefore, understanding the pathological and physiological mechanisms of IAs formation and rupture, comprehensively exploring potentially modifiable risk factors, and implementing strategies for preventing the formation and rupture of IAs are crucial in striving to offer better treatment options for patients.

Firstly, the occurrence of intracranial aneurysm (IAs) rupture is a clinical event influenced by various risk factors. Among them, the biological characteristics of IAs themselves are a crucial factor that cannot be ignored. Previous studies have identified several factors associated with endogenous IAs rupture, such as aneurysm size, location, and shape^16^. Over the years, clinical neurosurgeons have emphasized and considered size as a key indicator for assessing the instability and rupture risk of IAs. Consistent results from various studies indicate that a larger aneurysm size is a significant risk factor for aneurysm rupture^15,18^. Reports on the annual rupture rates of IAs in different diameter ranges internationally show rates of 1.2%, 3.1%, and 8.6% for diameters of 7–12 mm, 13–24 mm, and > 25 mm, respectively. Some studies suggest that 10 mm is an important threshold for assessing the risk of IAs rupture^17,19–24^.

Secondly, the mechanisms underlying the rupture of intracranial aneurysms (IAs) are complex and multifaceted, involving mechanical damage and dysfunction of the vascular wall due to chronic inflammation^25^. Endothelial dysfunction, primarily driven by oxidative stress, is considered a critical initial step in IA formation^26^. This dysfunction can be exacerbated by conventional risk factors, leading to abnormal activation of various inflammatory cells and mediators, which triggers a vascular inflammatory cascade that ultimately compromises the vascular wall structure. In the context of abnormal intracranial pressure, this cascade contributes to the formation of the local anatomical basis for IA development.

Studies have indicated that plasma homocysteine (Hcy) acts as an inflammatory molecule, and its elevation can damage the vascular endothelium and induce inflammation^27^. Hcy is also linked to the development of atherosclerosis^28^. Clinical studies and cell culture experiments have demonstrated that Hcy is toxic to all layers of the vascular wall, including the intima, media, and adventitia^29,30^. The mechanisms through which Hcy affects the vascular wall may involve disrupting endothelial homeostasis. Endothelial cells, which form the innermost layer of the vascular wall, are crucial for regulating vascular tone, promoting platelet aggregation and coagulation, maintaining smooth blood flow, and preventing thrombosis^31–33^. Hcy impairs endothelial cell function by disrupting the production of nitric oxide and destabilizing cerebral vessels. Additionally, cell culture studies reveal that Hcy induces endothelial cell inflammation by releasing inflammatory factors^34–36^.

Vascular smooth muscle cells (SMCs), the predominant cell type in the blood vessel wall, are essential for maintaining vascular tone and regulating blood pressure. Hcy-induced SMC proliferation disrupts collagen synthesis and extracellular matrix remodeling, leading to excessive degradation of the internal elastic layer and subsequent vascular remodeling^37–40^. Buemi et al.^41^ conducted animal experiments showing that short-term exposure to high levels of Hcy induces significant SMC proliferation in human and rat aortas, akin to the pathological SMC phenotype observed in atherosclerosis.

The adventitia, the outermost layer of the blood vessel wall, serves as a physiological barrier that maintains environmental balance within the vessel. Signals from the adventitia and surrounding cells play a critical role in vascular development, physiology, arterial wall remodeling, immune surveillance, and the regulation of vascular diseases^42^. Research by Canada et al.^43^ suggests that inflammation of the vascular wall may originate from the adventitia, though the precise mechanism remains unclear. Some hypotheses propose that elevated Hcy recruits inflammatory cells to the adventitia, leading to increased protein kinase activity^44^. Furthermore, Yao et al.^45^ observed in a rat model of carotid balloon injury that hyperhomocysteinemia (HHcy) increases collagen deposition and hyperplasia in the adventitia.

The hypothesis that elevated homocysteine (Hcy) levels may lead to vascular diseases was first proposed in 1969^46^. The high incidence of vascular complications in patients with severe hyperhomocysteinemia (HHcy) has raised concerns about its potential role in atherosclerosis and inflammation, suggesting its possible association with the occurrence and rupture of intracranial aneurysms (IAs). A series of basic experiments and observational clinical studies have shown a close correlation between HHcy and a high incidence of aneurysms^47–50^. However, there is currently no consistent consensus on whether Hcy increases the risk of intracranial artery rupture. Qiu et al.’s study indicated significantly higher plasma Hcy levels in patients with aneurysmal subarachnoid hemorrhage (SAH) compared to non-aneurysmal SAH patients, providing theoretical support for further exploration^51^.

Current research provides the latest perspectives on the risk, rupture trends, and preventive treatment of IAs associated with Hcy. From this standpoint, our retrospective study conducted in the Chinese population suggests that within the critical range of plasma Hcy levels (8.9 μmol/L to 15 μmol/L), various statistical methods consistently show a significant and independent correlation between critical Hcy levels and the rupture of small to medium-sized IAs, with statistically significant differences (*P* < 0.05).

In conclusion, with advances in imaging technology and increased awareness through population screening, as more cases are detected in the early stages, future research should focus on evaluating the effectiveness of Hcy prevention in IA rupture, determining the optimal timing for intervention, and balancing the risks and benefits of preventive treatment. This provides new insights into early prevention, reducing rupture risk, and improving outcomes for IAs.

## Data Availability

The original contributions presented in the study are included in the study, further inquiries can be directed to the corresponding authors.
